# Supplementary material for: Exploring the Dynamics of the TWIK-1 Channel
Source: Biophys J. 2016 Aug 23;111(4):775–84. doi: 10.1016/j.bpj.2016.07.009 (PMC5002071; doi:10.1016/j.bpj.2016.07.009)

Biophysical Journal, Volume *111*

Supplemental Information

Exploring the Dynamics of the TWIK-1 Channel

Victoria Oakes, Simone Furini, David Pryde, and Carmen Domene

SUPPLEMENTARY INFORMATION

Exploring the dynamics of the TWIK-1 channel

Victoria Oakes,[1] Simone Furini,[2] David Pryde,[3] Carmen Domene[1,4]

[1]Department of Chemistry, King's College London, Britannia House, 7 Trinity Street, London, SE1 1DB, UK.
[2]Department of Medical Biotechnologies, University of Siena, viale Mario Bracci 16, I-53100, Siena, Italy
[3]Worldwide Medicinal Chemistry, Pfizer Pfizer Neuroscience and Pain Research Unit, Portway Building, Granta Park, Great Abington, Cambridge CB21 6GS, UK
[4]Chemistry Research Laboratory, Mansfield Road, University of Oxford, Oxford, OX1 3TA, UK.

[1]Corresponding author: carmen.domene@kcl.ac.uk

Tel: +44 - (0) 2078483868

Figure S1. Root mean square deviation (RMSD) of the backbone of the transmembrane helices of TWIK-1 in simulations: (A) HSE, (B) HSEP, (C) HSD, (D) HSDP, (E) HSP and (F) HSPP.

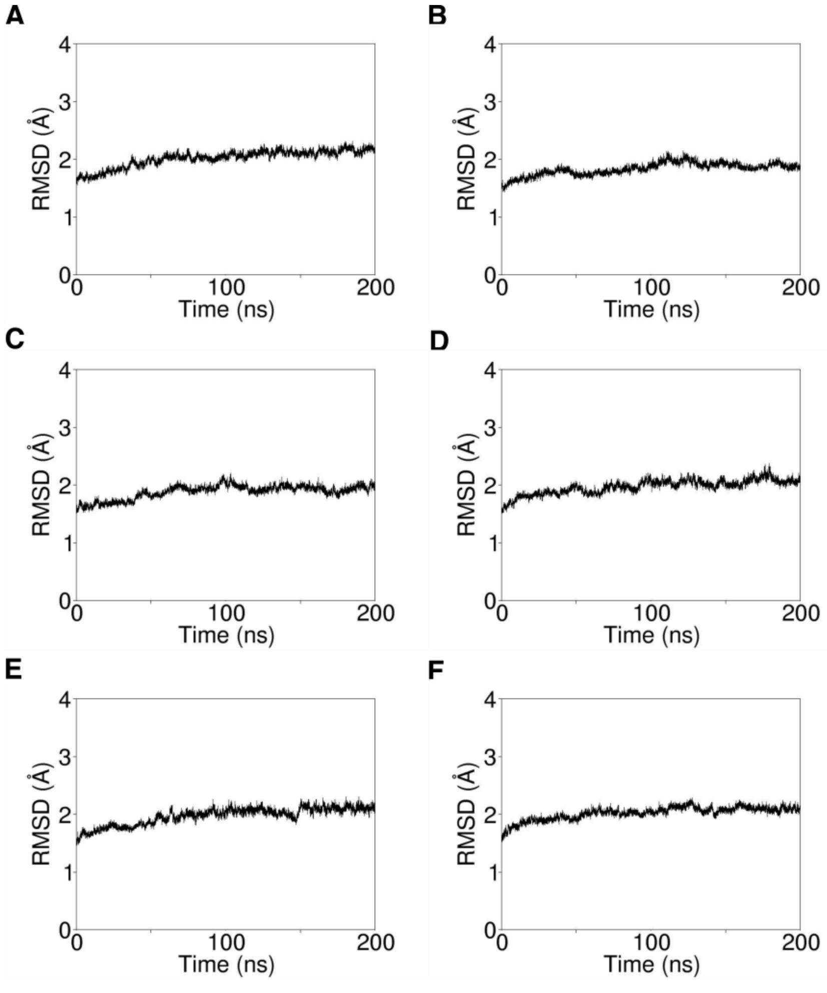

Figure S2. Root mean square deviation (RMSD) of the extracellular cap corresponding to residues S80 to D103 in simulations: (A) HSE, (B) HSEP, (C) HSD, (D) HSDP, (E) HSP and (F) HSPP.

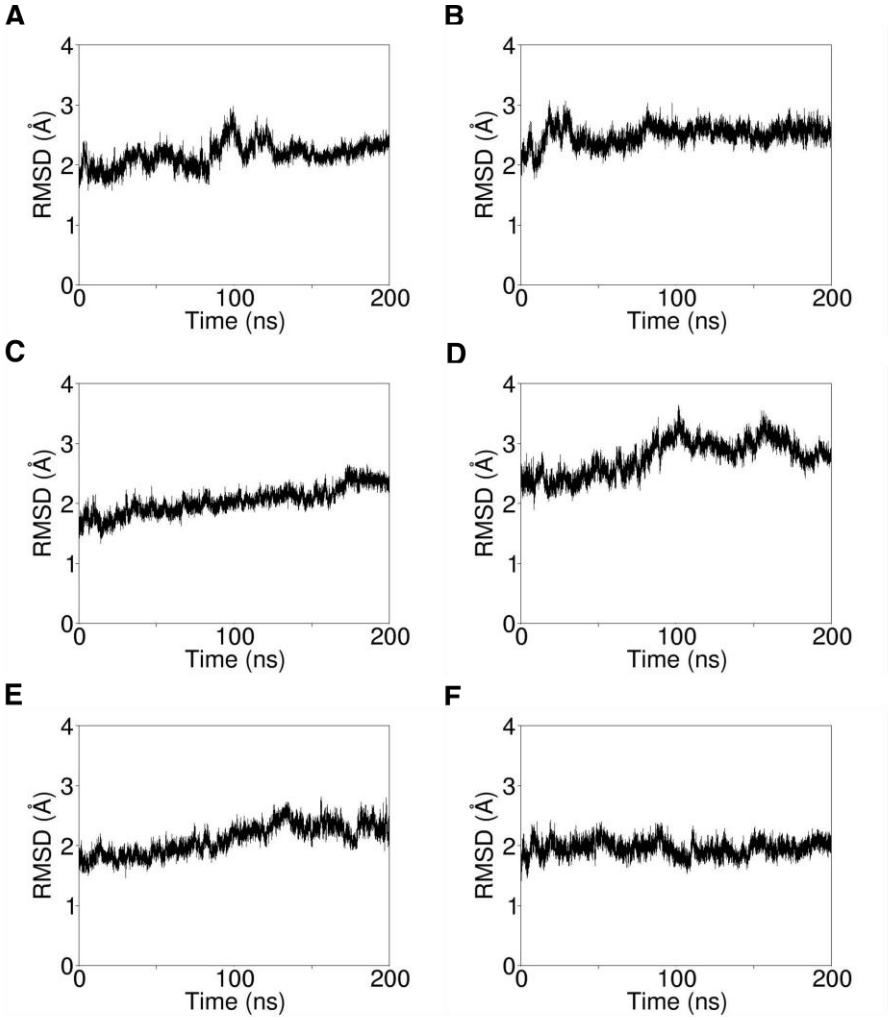

Figure S3. Representative snapshots of each ion configuration throughout simulations (A) HSE, (B) HSEP, (C) HSD, (D) HSDP, (E) HSP and (F) HSPP. The selectivity filter residues T117 to G121 and T225 to G229 are displayed in licorice representation, with Van der Waals spheres representing ions and water molecules, with oxygen, nitrogen, carbon, sodium and potassium atoms shown in red, blue, cyan, yellow and orange respectively.

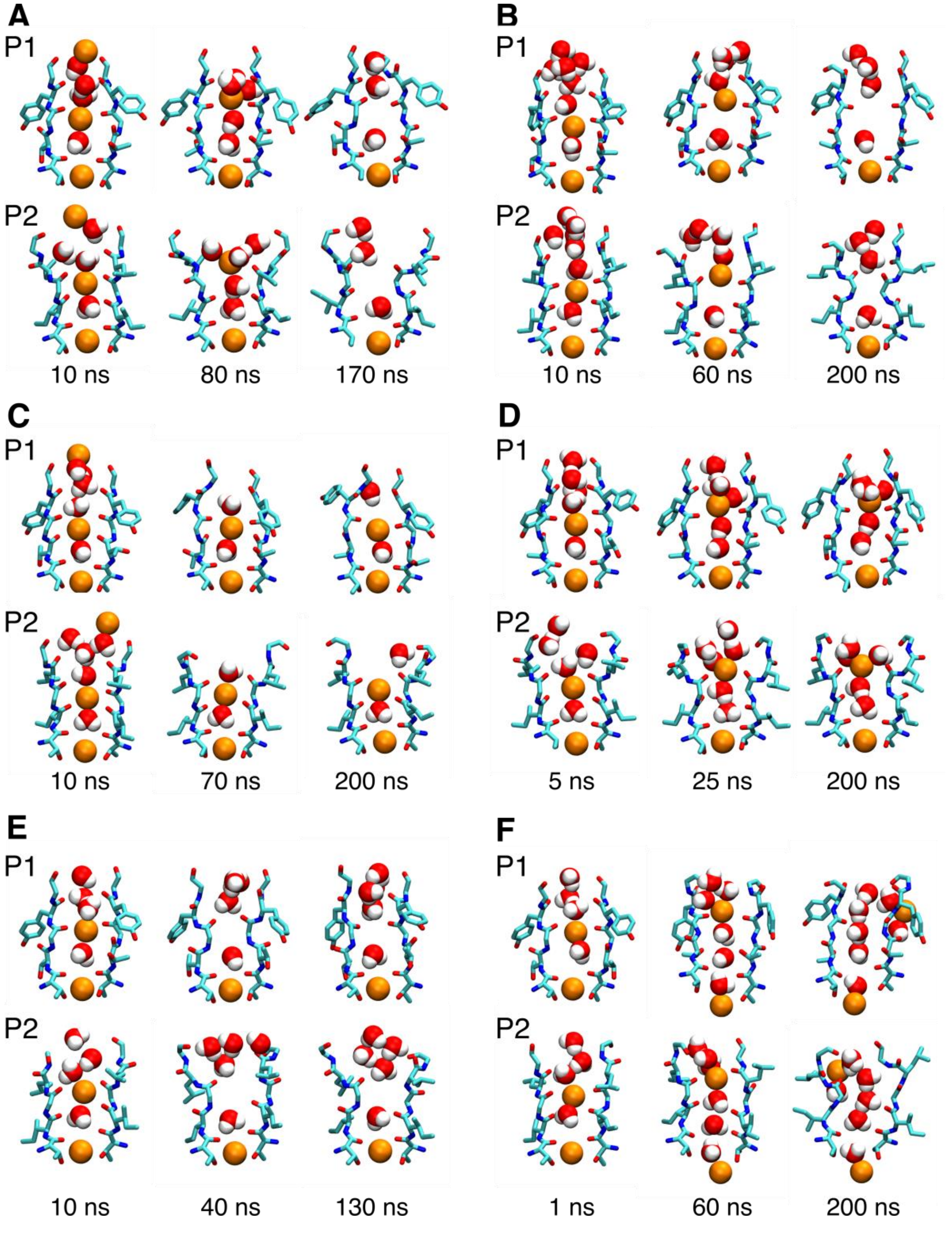

Supplement: Document S1. Figs. S1–S3 [file mmc1.pdf]
