# Supplementary material for: Exploring the Dynamics of the TWIK-1 Channel
Source: Biophys J. 2016 Aug 23;111(4):775–84. doi: 10.1016/j.bpj.2016.07.009 (PMC5002071; doi:10.1016/j.bpj.2016.07.009)

Biophysical Journal
Article

Exploring the Dynamics of the TWIK-1 Channel

Victoria Oakes,[1] Simone Furini,[2] David Pryde,[3] and Carmen Domene[1,4,*]
[1]Department of Chemistry, King's College London, London, United Kingdom; [2]Department of Medical Biotechnologies, University of Siena, Siena, Italy; [3]Worldwide Medicinal Chemistry, Pfizer Neuroscience and Pain Research Unit, Cambridge, United Kingdom; and [4]Chemistry Research Laboratory, Mansfield Road, University of Oxford, Oxford, United Kingdom

ABSTRACT Potassium channels in the two-pore domain family (K2P) have various structural attributes that differ from those of other K^+ channels, including a dimeric assembly constituted of nonidentical domains and an expansive extracellular cap. Crystallization of the prototypical K2P channel, TWIK-1, finally revealed the structure of these characteristics in atomic detail, allowing computational studies to be undertaken. In this study, we performed molecular-dynamics simulations for a cumulative time of ~1 μs to discern the mechanism of ion transport throughout TWIK-1. We observed the free passage of ions beneath the extracellular cap and identified multiple high-occupancy sites in close proximity to charged residues on the protein surface. Despite the overall topological similarity of the x-ray structure of the selectivity filter to other K^+ channels, the structure diverges significantly in molecular-dynamics simulations as a consequence of nonconserved residues in both pore domains contributing to the selectivity filter (T118 and L228). The behavior of such residues has been linked to channel inactivation and the phenomenon of dynamic selectivity, where TWIK-1 displays robust Na^+ inward flux in response to subphysiological K^+ concentrations.

INTRODUCTION

The two-pore domain K^+ family (K2P) forms a structurally and functionally distinct class of K^+ channels. These channels are responsible for background leak K^+ currents that stabilize the negative resting potential of the cell, and also play roles in ion homeostasis, hormone secretion, cell development, and excitability (1). K2P channels can be modulated by a vast array of regulatory stimuli, such as pH (2), temperature (3), mechanical stress (4), the presence of polyunsaturated fatty acids (5), and volatile anesthetics (6). The expression of these channels in the heart and brain has also led to increased exploration of their therapeutic potential for the treatment of various neuronal and cardiac disorders (5,7).

K2P channels assemble as a dimer of dimers, with each subunit containing four transmembrane helices (H1–H4) and two pore loops (P1 and P2). The recent crystallization of multiple K2P channels (TWIK-1 (8), TRAAK (9,10), and TREK-2 (11)) has greatly advanced our understanding of this unique architecture, exhibiting various conserved features throughout the family. For example, an extracellular cap (EC) between H1 and P1 is present, extending 35 Å above the transmembrane helices, with the apex of each subunit connected by a disulphide bond. An unrestricted pathway at the cytoplasmic entrance is also observed throughout, revealing apertures of comparable dimension to other K^+ channels that are considered open. This is in line with experimental studies suggesting that structural changes at or near the selectivity filter (SF) form the predominant gating mechanism across the K2P family, which shares its canonical structure with other K^+ channel families.

With regard to the electrophysiological properties of the K2P channels, in asymmetrical K^+ concentrations, almost all K2P subfamilies (TREK, TALK, TASK, THIK, and TRESK) conform to the typical properties of leak K^+ currents, demonstrating outward (or open) rectification. The functional properties of the TWIK family have remained elusive due to low levels of activity recorded in physiological K^+ gradients (12). This phenomenon was originally attributed to the sumoylation of a lysine residue in the C-terminal domain, which could be inactivated by a single-point mutation (13). However, this was later disproved as the primary mechanism of TWIK-1 silencing (14). The mutation of consecutive isoleucine residues in the C-terminal domain was found to induce strong expression of TWIK-1 in the cell membrane (14); however, meager currents were still recorded relative to other K2P channels in the same conditions (15,16). Such observations led to the proposal that the prohibition of ionic current is an inherent property of TWIK-1 (17). Furthermore, reports have suggested that TWIK-1 does not exhibit such open rectification. Various mutant

Submitted December 30, 2015, and accepted for publication July 11, 2016.
*Correspondence: carmen.domene@kcl.ac.uk
Editor: Emad Tajkhorshid.
http://dx.doi.org/10.1016/j.bpj.2016.07.009
© 2016 Biophysical Society.
This is an open access article under the CC BY license (http://creativecommons.org/licenses/by/4.0/).
CrossMark

channels were found to both increase the conductance and shift the rectification properties to those of other K2P channels, indicating a convoluted regulation mechanism.

In particular, TWIK-1 was the first channel to display variable selectivity in response to external stimuli such as lowered K^+ concentrations (18), increased NH_4^+ and Rb^+ concentrations (19), and acidification (20). Hypokalemia (when extracellular K^+ concentrations are lower than 3.5 mM) is observed in up to 20% of hospitalized patients and has been associated with an increased risk of sudden cardiac arrest (21). The adjusted selectivity of TWIK-1 in these conditions indicates the presence of an inward Na^+ current in response to the Na^+ concentration gradient, and this property is known to depolarize cardiomyocytes and potentially contribute to cardiac arrhythmia (7,22). As a consequence, TWIK-1 has emerged as a putative drug target for antiarrhythmic drugs (23). In-depth exploration of the molecular determinants of conduction, selectivity, and gating in TWIK-1 will likely contribute to the development of targeted therapies.

Previous molecular-dynamics (MD) studies of TWIK-1 identified a hydrophobic cuff in the inner pore that is responsible for a cyclical dewetting process and consequently an unfavorable barrier to conduction (24). This effect was suggested to be influenced by the presence of lipid molecules in proximal fenestrations (25), and was supported by studies involving gain-of-function mutagenesis of such residues to hydrophilic components (24). However, the behavior of the SF and its implications for the mechanism of conduction, selectivity, and gating have not yet been examined. Therefore, we conducted an MD study utilizing the crystal structure of TWIK-1 to obtain insight into these phenomena.

MATERIALS AND METHODS

System setup

The crystal structure of TWIK-1 was retrieved from the Protein Data Bank (PDB: 3UKM) at a resolution of 3.4 Å (residues 19–288) (8). Five potassium ions were resolved in the crystal structure in the internal SF sites (S1–S4) and S0, suggesting that the structure is representative of an open, conductive state. Three of these ions were kept in positions S0, S2, and S4, and those in positions S1 and S3 were converted to water molecules to represent one of the low-energy conformations identified in previous studies (26). Crystallographic waters were kept. Missing loops were modeled using Modloop (27) and combined with the crystal structure. N- and C-termini were acetylated and methylated, respectively. Residues C69 of opposing subunits were linked by a disulfide bond. Default protonation states were used for ionizable residues, supported by PropKa calculations (28). SOLVATE1.0 was used to solvate the protein and fill cavities present in the structure. A preequilibrated lipid bilayer of 1-palmitoyl-2-oleoyl-*sn*-glycero-3-phosphocholine (POPC) molecules was used. The protein was aligned to the bilayer normal and inserted into the membrane. All lipid molecules within 1.2 Å of protein atoms were removed. The combined system was then solvated to produce a rectangular water box of dimensions $93 \times 93 \times 118$ Å. Potassium and chloride ions were added to neutralize the system to a biologically relevant ion concentration (150 mM) using the Autoionize Plugin of VMD (29). All water molecules deemed as overlapping (distance < 1.2 Å) with the protein, lipids, and ions were removed, resulting in a system size of ~90,000 atoms. Trajectories of 200 ns were produced and denoted as HSE (δ position in H122 protonated, D230 unprotonated), HSEP (δ position in H122 protonated, D230 protonated), HSD (ε position in H122 protonated, D230 unprotonated), HSDP (ε position in H122 protonated, D230 protonated), HSP (both δ and ε positions in H122 protonated, D230 unprotonated), and HSPP (both δ and ε positions in H122 protonated, D230 protonated).

MD simulations

NAMD2.9 was employed to calculate trajectories (30). We used the CHARMM36 force field for the protein, CHARMM36 for lipids (31), the TIP3P model for water (32), and the CHARMM NBFIX parameters for ions (33,34). The particle mesh Ewald method was used for the treatment of periodic electrostatic interactions, with an upper threshold of 1 Å for grid spacing (35). Electrostatic and van der Waals forces were calculated every time step. A cutoff distance of 12 Å was used for van der Waals forces. A switching distance of 10 Å was chosen to smoothly truncate the nonbonded interactions. Only atoms in a Verlet pair list with a cutoff distance of 13.5 Å (reassigned every 20 steps) were considered (36). The SETTLE algorithm was used to constrain all bonds involving hydrogen atoms, to allow the use of a 2 fs time step throughout the simulation (37). The Nose-Hoover-Langevin piston method was employed to control the pressure with a 200 fs period, 50 fs damping constant, and a desired value of 1 atmosphere (38,39). The system was coupled to a Langevin thermostat to sustain a temperature of 310 K throughout, to maintain the model membrane above its gel transition temperature.

Equilibration protocol

The systems were subjected to 1000 steps of minimization and equilibrated for a total of 3.5 ns. The duration of each equilibration step was 500 ps with a gradual reduction of restraints throughout: 1) protein atoms, ions in the SF, lipid headgroups, and water molecules within protein cavities restrained; 2) protein atoms, ions in the SF, and water molecules within protein cavities restrained; 3) protein atoms and ions in the SF restrained; 4) protein backbone atoms, SF atoms, and ions in the SF restrained; 5) SF atoms and ions in the SF restrained; 6) SF backbone atoms and ions in the SF restrained; and 7) SF ions restrained only.

RESULTS AND DISCUSSION

K2P channels exhibit a unique architecture (illustrated in Fig. 1 *A*) formed from the assembly of two identical subunits (denoted A and B). Each subunit consists of two nonidentical pore domains, with the former including an expansive EC. Despite the substantial structural variations, TWIK-1 displays the archetypal conductive SF structure, which is highly conserved in numerous human and bacterial K^+-channel crystal structure analogs (Fig. 2 *A*). Backbone carbonyls from each subunit and pore domain within the subunits assemble in a cage-like structure to form four adjacent binding sites (S1–S4) that are capable of binding dehydrated K^+ ions. Additional sites are capable of binding partially hydrated species at the intracellular (SC) and extracellular (S0) exits. The sequence is divergent from the signature sequence TXGYG, where X represents any hydrophobic amino acid; P1 is TTGYG and P2 is TIGLG. On closer inspection, a degree of asymmetry is observed in the pore; the distance between the T118 (P1) carbonyls is

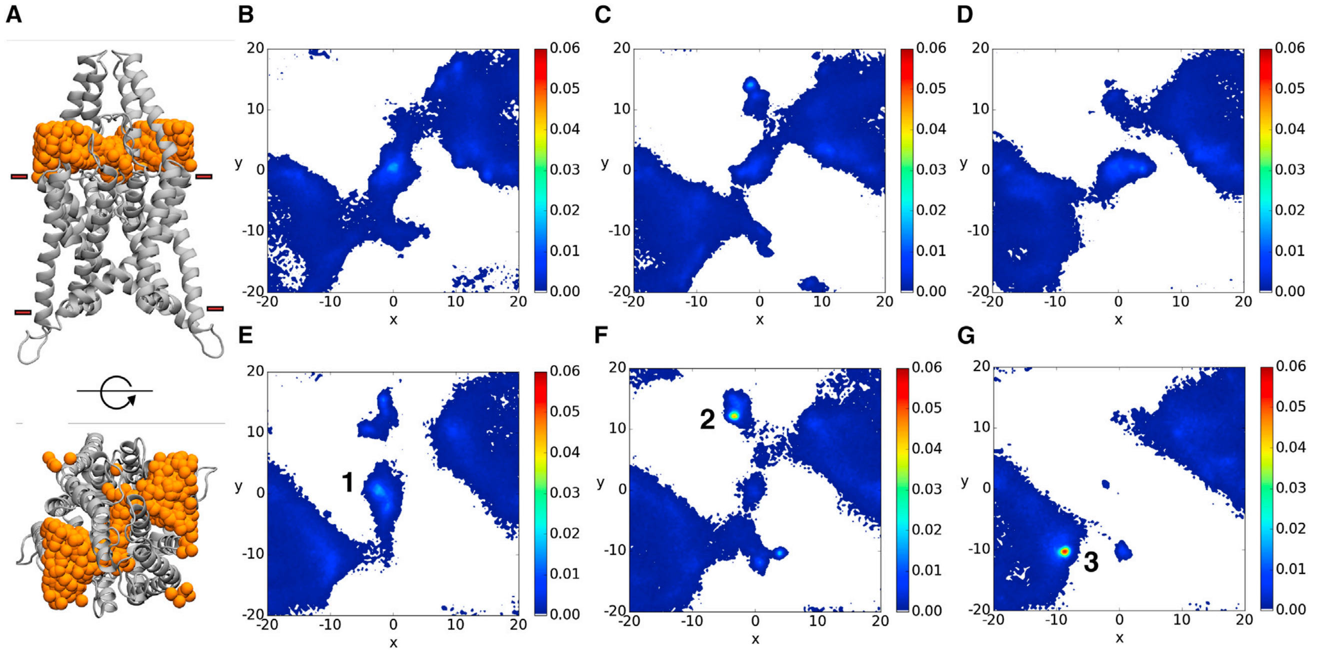

FIGURE 1 Extracellular ion transport pathways in TWIK-1. (*A*) Side view (*upper panel*) and top view (*bottom panel*) of the region between the EC (G80) and the SF (G121), where the position of K^+ ions (*orange spheres*) was tracked. (*B–G*) Color maps showing the position of extracellular ions entering the filter region in simulations HSE, HSD, HSP, HSEP, HSDP, and HSPP, respectively. Ion positions were measured as the *x* and *y* coordinates of the center of mass and then discretized into bins of 0.5 Å. To see this figure in color, go online.

4.3 Å, compared with 4.8 Å between I226 (P2) atoms. Furthermore, the SF is known to be influenced by external pH, with ionizable residues present at the top of the SF in both P1 and P2. H122, in particular, has been established as the putative proton sensor in TWIK-1, as well as in the K2P channels TASK-1 and TASK-3, that responds to changes in extracellular pH (17). How these features influence the dynamics of the SF and hence ion permeation on

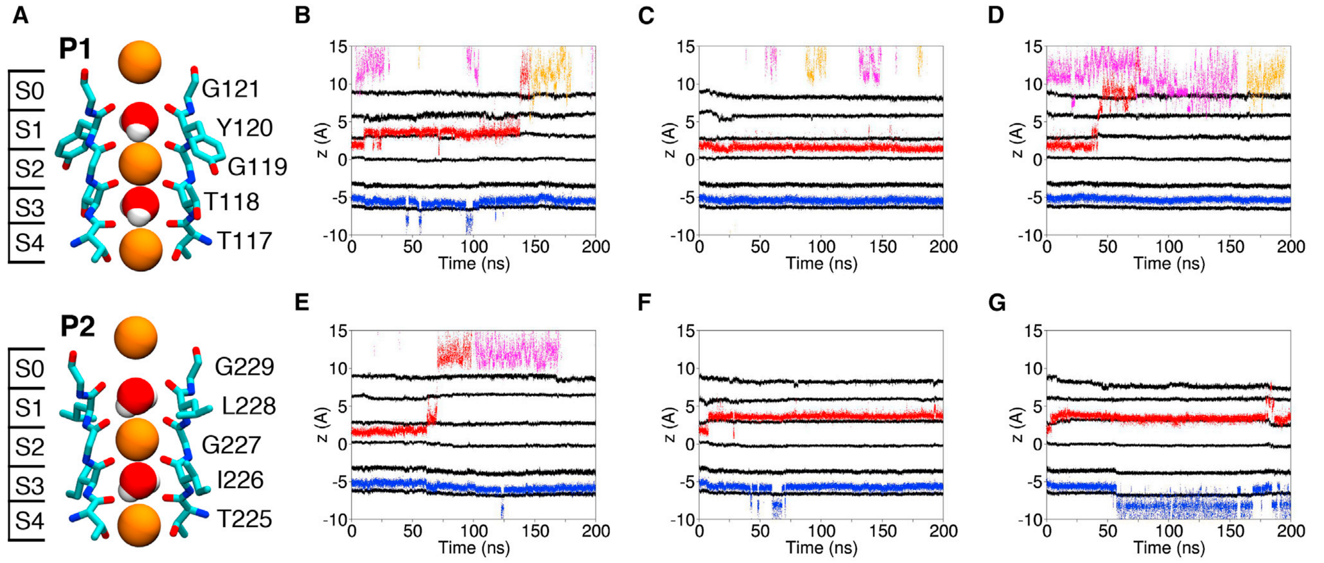

FIGURE 2 (*A*) Crystal structure of the P1 and P2 domains of the TWIK-1 SF, with the initial ion configuration used in all simulations. Definitions of the binding sites are provided on the left. The SF residues T117–G121 and T225–G229 are displayed in licorice representation, with van der Waals spheres representing ions and water molecules. Oxygen, nitrogen, carbon, sodium, and potassium atoms are shown in red, blue, cyan, yellow, and orange, respectively. (*B–G*) Ion trajectories in simulations HSE (*B*), HSD (*C*), HSP (*D*), HSEP (*E*), HSDP (*F*), and HSPP (*G*). The black traces correspond to the center of mass of the oxygen atoms of the SF residues that contribute to the binding sites. The blue, red, yellow, and pink trajectories correspond to the trajectories of individual K^+ ions. Representative snapshots of each ion configuration can be found in Fig. S3. To see this figure in color, go online.

an atomistic level is currently unknown. To address this issue, we performed multiple independent MD simulations to gain insight into the properties of the TWIK-1 SF, as outlined in Materials and Methods.

In all systems, the root mean-square deviations of the transmembrane domain (Fig. S1 in the Supporting Material) and the EC (Fig. S2) show initial jumps associated with the release of restraints present in the initial equilibration procedure, and remain under 2.5 Å and 3.5 Å, respectively, throughout, suggesting that the channel is stable and representative of the determined structure. It is well established in the literature that cytoplasmic gating does not modulate activity in K2P channels. In agreement with this, the cytoplasmic gate does not undergo any significant constriction throughout our simulations, and the slide helix remains parallel to the membrane normal.

Ion transport to the SF

K2P channels possess an extracellular domain that is distinct from other K^+ channel families, extending ~35 Å above the transmembrane domain. The geometry of this region imposes obvious steric constraints on the diffusion pathway to the central pore (Fig. 1 *A*). In addition, the residual composition is predominantly negatively charged, providing a sink for surrounding cations. To gain insight into the transport of ions in this region, we tracked the x and y positions of ions entering the region between the SF and the turret above, excluding occupancy of the S0–S4 sites. The bounding box was defined by z coordinates of the Cα atoms of G80 in the EC and residue G121 at the mouth of the SF, with the x-y coordinates restricted by the region occupied by the transmembrane helices. Density plots of ion distribution, displayed in Fig. 1, *B–F*, show that bidirectional diffusion of ions occurs through side portals of the protruding domain. Negligible diffusion is seen in Fig. 1 *G*, corresponding to the HSPP simulation in which both H122 and D230 are protonated.

The density plots in Fig. 1 pinpoint three unique regions of increased ion density. First, the region above the SF, denoted 1 in Fig. 1 *E*, is consistently a high-occupancy region, with the exception of the HSPP simulation. The charged nature of the SF and above extracellular helices, in addition to contacts with surrounding residues, all contribute to binding in this region, with subtle differences dependent on the filter conformation. A central site can be occupied between the carbonyls of G121 and G229 in both subunits, at the upper bounds of the S0 site. Additional instances are also observed in which ions can be found in off-axis sites interacting with H122 (protonated in the δ or ε position), G121, G229, D230 (unprotonated), S86 (EC), or N87 (EC) residues.

A site was identified in the HSDP simulation (labeled 2 in Fig. 1 *F*) occupying a region directly behind the P1 SF sequence, with ions predominantly interacting with N101, H122, N242, and E235. An additional site in close proximity to this (denoted 3 in Fig. 1 *G*) was also found in the HSPP simulation. This site is present between the P1 SF sequence and P-loop helix, and is defined by direct interactions with E207 (H3-P2 loop), V232 (P2-H4 loop), E235 (P2-H4 loop), and extracellular water molecules. The close proximity of these sites to the SF may have implications for the conformation of the SF and hence ion permeation.

Ion-binding sites in the SF

To probe the behavior of ions within the SF, we tracked the positions of the four ions in contact with the SF for the longest period of time (Fig. 2, *B–G*) and analyzed their behavior. No complete permeation events were observed within any trajectory. However, individual ion movements provide insight into the stability of the K^+ binding sites and the behavior of ions within them. A comparison of the conduction profiles of all of the simulations reveals that the S2–S4 K^+ configuration, as well as the structure observed in the crystallographic data, shows variable stability in the TWIK-1 SF and is sustained for ~5–200 ns. This is in spite of the absence of a concentration gradient or an applied voltage.

With the exception of HSPP, the ion occupying the S4 site is generally stagnant for the timescale of our simulations in the absence of additional ions, due to the consistent coordination to backbone carbonyls and side-chain hydroxyl groups of T117 and T225, resulting in a coordination number of 7 or 8 throughout. In contrast, the ion that originally occupies S2 is subject to reduced coordination at both the upper and lower bounds of the site, and abstraction from this site is observed in all simulations except HSD. T118 in P1 shows increased conformational freedom, resulting in prolonged periods where ion contacts cannot be formed and hence elevation of the ion in the site, which consequently reduces the coordinating ability of I226 in the equivalent position in P2. Additionally, structural changes originating from the top of the P2 SF sequence also lead to reduced coordination of G227, further contributing to the instability of the occupying ion. In the case of HSD, conformational changes at the top of the P1 domain of the SF result in a constriction at the S0 site and the removal of multiple carbonyls from the S1 site, rendering the S2 site the most favorable for an ion in this region of the SF. Qualitatively, these observations are in agreement with the crystallographic data, which demonstrate ion density in S1 to S4 sites.

In the HSE, HSDP, and HSPP simulations, the S1–S4 conformation is sustained for ~100–200 ns. Due to the increased conformational freedom of the upper region of the filter in both the P1 and P2 domains, the ion in S1 is subject to coordination by carbonyls from two G119 residues. The remaining contributions come from Y120 and G227 residues and additional water molecules depending on the conformation of the SF, which generally exhibits a full

coordination shell despite divergence from the canonical structure. Furthermore, transient constrictions in the S0 region of the P1 domain, similar to those observed in the HSD simulation, likely contribute to ion maintenance in this site, and expulsion to the extracellular solution is only permitted in the absence of these constrictions.

Structure of the SF

Experimental evidence indicates that the K^+ channel SF can adopt multiple conformations in addition to the stereotypical conductive conformation (40). Certain voltage-gated K^+ channels have shown Na^+ conductance in the absence of internal K^+ ions, suggesting that the SF can adopt different open conformations with shifting selectivity (41–46). In the case of TWIK-1, inward rectification of Na^+ and outward rectification of K^+ have been measured in low extracellular K^+ concentrations and acidic conditions, even though it exhibits the attributes of a highly selective channel in normal physiological conditions (18), albeit with low conductance properties. These results indicate that TWIK-1 has at least three distinct SF conformations: one conductive, one inactivated, and one with altered selectivity properties. Therefore, we focused our attention on characterizing the conformations of the TWIK-1 SF in response to the range of ionic configurations we identified.

The crystal structure is representative of a typical conductive conformation that consists of four contiguous binding sites and is capable of providing a full coordination shell to dehydrated K^+ ions (47). This structure is observed initially in the MD trajectories (Fig. 3 *A*) but diverges rapidly in all simulations to a number of conformations that contain defective coordination sites yet maintain the general framework of the SF and can be considered partially conductive. This is due to an amalgamation of distinctive P1 and P2 conformations (Fig. 3 *B*). The disparity compared with the typical structure in P1 is localized in the region of T118, which exhibits rotational freedom, causing lateral expansion of the S2 and S3 sites. Crystallization of the noninactivating E71A KcsA mutant revealed remarkable similarities to the structure of P1 in this region (48). In the P2 domain, the canonical structure is lost in the upper region, with residue D230 exhibiting a dynamic behavior, both in protonated and unprotonated forms. A consequence of this higher mobility is a reduced ion coordination by protein atoms in binding sites S0–S2. The functional state of this conformation cannot be attained in our simulations, but as all sites remain viable to accommodate K^+ ions, it is likely that conduction, even if less favorable, can still occur in this state. This is observed in all simulations except HSD, where a constriction in the S0 site is observed in the P1 domain (Fig. 3 *C*), occluding water and ions from entering the SF from this angle for the remainder of the simulation. The predominance of such states throughout the simulation may have implications for the low conductance properties of TWIK-1 relative to other K2P channels.

The vacancy of both the central S2 and S3 sites induces conformations in which the S2 site is physically occluded by backbone rearrangements of SF residues (Fig. 3 *D*). In the HSE and HSP simulations, this occurs by movement of T118 and G119 in P1, whereas in the HSEP and HSPP simulations, this state is observed in I226 and G227 in P2. In these configurations, ions and water molecules are excluded from the S2 site for the remainder of the simulation. The S4 and S3 sites are maintained, with diffuse S0 and S1 sites containing multiple molecules as in the previous conformation. The constriction of the filter shares similar attributes with the crystal structure of KcsA in low K^+ concentrations, where the SF is blocked by a constriction involving V76 and G77 residues, and V76 is oriented away from its optimal position (49). This was confirmed to be nonconductive (50–52) and suggested to be representative of a C-type inactivated state (53). It must be noted that this state is stable on a millisecond timescale, which is unattainable during our simulations; therefore, it is possible that this conformation is an intermediary state that can block

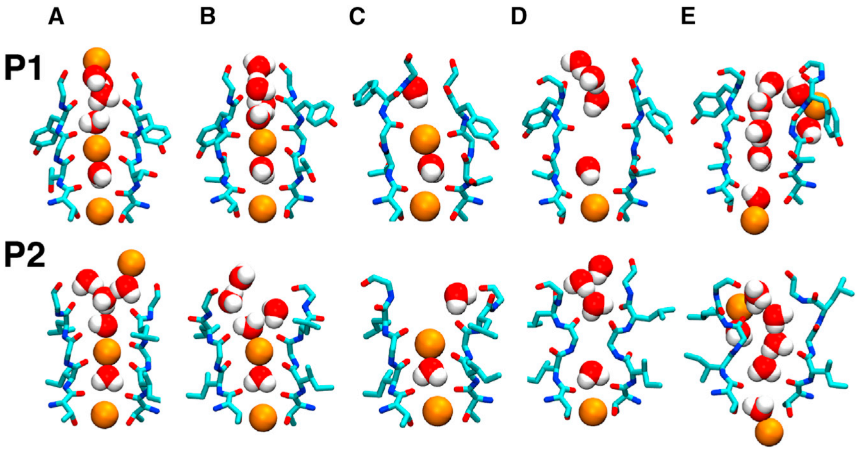

FIGURE 3 Snapshots of the identified SF conformations. The SF residues T117–G121 and T225–G229 are displayed in licorice representation, with van der Waals spheres representing ions and water molecules, and oxygen, nitrogen, carbon, sodium, and potassium atoms shown in red, blue, cyan, yellow, and orange, respectively. (*A*) HSD, 10 ns. (*B*) HSDP, 5 ns. (*C*) HSD, 200 ns. (*D*) HSEP, 200 ns. (*E*) HSPP, 200 ns. To see this figure in color, go online.

permeation transiently, but does not represent the typical inactivated state.

Finally, in the HSPP simulation, a novel, to our knowledge, structure of the SF was observed in which all binding sites were depleted of ions but the SF remained open (Fig. 3 *E*). Previous computational studies suggested the existence of a pathway behind the SF (54) for water transport in collapsed K^+ channels (55). However, these results demonstrate an atomistic representation of the K^+ SF that can stably occupy water in the absence of ions and potentially allow water permeation directly through the SF. These observations provide insight into the unique behavior of the SF of TWIK-1 as a consequence of a single sequence difference in each domain.

Structural changes in P1

Compelling experimental evidence suggests that the hydrogen (H)-bond network behind the SF plays an integral role in determining the structure of the SF, influenced by the presence of structural water molecules and the orientation of local residues (56). Therefore, we analyzed the atomic interactions that stabilized the observed conformations. An examination of T118 is of particular importance due to its confirmed role in the variable selectivity of TWIK-1 in response to low extracellular K^+ concentrations (18). To understand the interrelation between the behavior of this residue and the ionic configurations, we characterized its conformation throughout our simulations using the Ψ (backbone) and X (side chain) dihedral angles (Fig. 4, *A* and *B*). Overall, the Ψ backbone conformations could be prorated into seven clusters, with Ψ values centered at approximately −95°, −45°, 20°, 65°, 110°, 140°, and 170°. These clusters were denoted I–VII in ascending order. In each cluster, the side-chain dihedral angle X could occupy three rotameric states, g+, g−, and t, corresponding to average X angles of 36°, −41°, and −176°, respectively (57). These conformations will be referred to by these classifications henceforth, and the structures of the most highly populated conformations are shown in Fig. 4 *B*.

Conformation II (g−) is representative of the crystal structure with the carbonyls poised for ion binding, and therefore can be considered to be conductive. This conformation is one of the least populated conformations in our simulations, displaying a complex H-bond network as a means of stabilization for a fully conductive filter. T118 and G119 act as H-bond donors to the T113 carbonyl on the pore helix, and the T118 side chain is capable of H-bonding with both S116 and S222 side chains. The remaining filter residues interact with up to two structured water molecules, which in turn contact the side chains of T113, H122, and T123.

In the additional T118 conformations we identified, the backbone carbonyl deviates from this orientation; hence, the extent to which T118 can bind permeant ions in S2 and S3 is compromised. Conformation IV (g−) represents a flipped state, with the carbonyl perpendicular to the pore axis. Further rotation of T118 results in a blocked conformation, V (g−). Backbone atoms in this region physically occlude the permeation axis, resulting in complete removal of the S2 site and blockage of the SF. Such conformations have been established as part of the normal functioning of K^+ channels via MD simulations, depending on the ion occupation of the SF. The H-bond network is highly conserved with regard to conformation II (g−), with minimal reorientations involving the T113 carbonyl and water molecules that now interact with the T118 backbone. In conformation III (t), the carbonyl is rotated ~75° relative to the crystal structure, displaying an H-bond between the side-chain hydroxyl and backbone carbonyl of T118, and

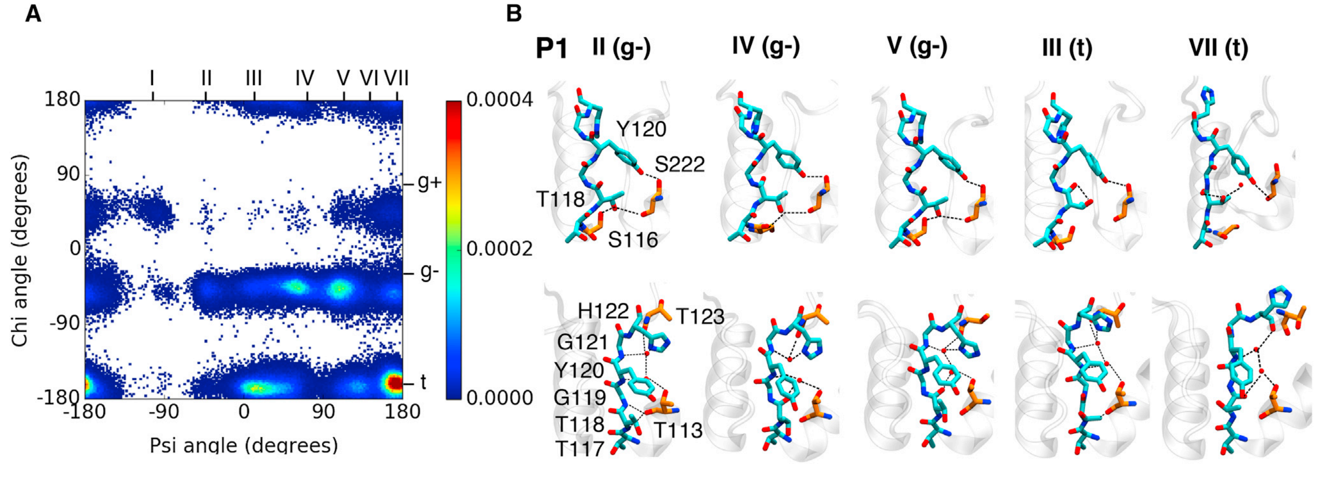

FIGURE 4 (*A*) Heat map of Ψ and X angles of T118 throughout all simulations. (*B*) Snapshots of the full P1 SF in the most populated T118 conformations (from left to right): II (g−)/conductive, IV (g−)/flipped, V (g−)/inactivated, III (t)/open conformation (I), and VII (t)/open conformation with H122 in the up state. The upper and lower panels represent the front and side views of the SF, respectively. SF residues T117–H122 and H-bond partner residues S113, T123, and S222 are shown in cyan and orange, respectively, with red spheres representing water molecules. To see this figure in color, go online.

conserved H-bonding properties elsewhere. As hydrophobic residues usually occupy this position, this conformation appears to be unique to TWIK-1 and, to the best of our knowledge, has not been characterized in previous computational studies.

A prerequisite of the H-bond network described in these conformations is that H122 must be in a so-called down state, where the histidine ring is embedded in the small cavity behind the SF. It must be noted, therefore, that H122 can also occupy an up state, with the ring displaying enhanced flexibility above the filter. In this case, extracellular water molecules penetrate the region previously occupied by the H122 side chain, and interact with selectivity filter residues to stabilize similar conformations. An additional conformation is also observed in which T118 is in the region of the Ramachandran plot corresponding to a β-sheet, with the T118 side chain occupying the pore and H-bonding with surrounding water molecules. In this case, H122 is primarily in the up state, and is predominant throughout our simulations.

In both conformations III (t) and VII (t), coordinating atoms in the S2 and S3 sites are removed, providing a direct link between the behavior of T118 and the structure of the TWIK-1 SF. The S2 site has been identified as the most selective site in K^+ channels; therefore, these conformations may represent a potentially nonselective state, as shown experimentally in reduced K^+ concentrations and extracellular pH (18,20). The importance of the so-called pH sensor, H122, for the different conformations of P1 suggests a further association between the latter stimulus and the dynamic selectivity phenomenon.

Structural changes in P2

The P2 sequence in TWIK-1 is also not conserved with respect to other K^+ channels, since the representative TXGYG sequence is constituted of TIGLG in this channel. The sampled conformations of this domain display a variable H-bond network involving water molecules and pore helix (Y217 and I221), SF (I226, G227, and G229), and loop (D230 and Y231) residues. Favorable hydrophobic interactions between the bulky side chains of I226 and L228 can also be identified. The lower region of the P2 filter domain (S2–S4) is highly conserved with respect to other K^+ channels and thus exhibits paramount stability relative to P1, demonstrating only conductive and constricted conformations. With regard to the upper region of the filter, the preservation of the S0–S2 sites is dependent on the maintenance of interactions between Y217 and D230 (Fig. 5). Detachment of these residues (in both protonation states) propagates structural changes throughout the SF, notably rotation of L228 and G229, resulting in numerous structural variants in which the upper sites are removed and incapable of ion binding. Most commonly, the behavior of this region is largely determined by dynamic interactions

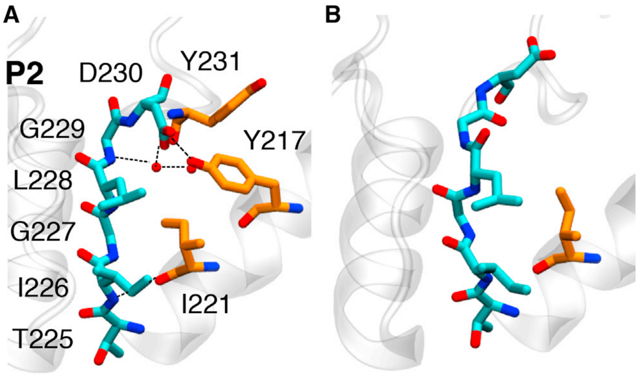

FIGURE 5 Snapshots of the observed P2 conformations in licorice representation, using the coloring scheme defined in Fig. 2 for P2 residues, and the carbon atoms of additional H-bonding residues shown in orange. (*A*) Conductive conformation. (*B*) Conformation with S2–S4 sites maintained, with disruption in the upper sites. To see this figure in color, go online.

with surrounding water molecules and cations, as well as transient interactions with K131.

Comparison with other K^+ channels

Investigations of SF dynamics have focused largely on the prokaryotic K^+ channel, KcsA. The crystal structure of KcsA exhibits a complex network of H-bonds involving contributions from the SF (G77, Y78, and G79), adjacent pore helices (W67 and E71), the succeeding extracellular loop (D80), and an adjacent water molecule, stabilizing the conductive conformation of the filter (49). In the nonconductive case, a reorganized H-bond arrangement was observed that was devoid of direct E71-Y78 and E71-G77 interactions, which were replaced by three structured water molecules behind the SF, the presence of which is thought to control the recovery from slow inactivation (58). A multitude of structural, functional, and computational investigations have since indicated that the W67, E71, D80 triad is crucial in determining the degree to which C-type inactivation occurs (48,59,60). Crystal structures of the E71A mutant provided an atomistic description of a noninactivating SF. The nonflipped state showed remarkable similarities to the conductive state of KcsA in spite of low K^+ concentrations. Remarkably, the W67-D80 interaction was maintained. In contrast, W67 and D80 occupy alternative conformational states in the flipped state, with the latter extending above the SF, resulting in a novel, to our knowledge, SF framework in which the S2 and S3 sites are merged due to rotations of V76.

Residues that occupy equivalent positions in TWIK-1 play an integral role in the conformations we observed, despite their low conservation. In P1, T113 and H122 occupy the positions of E71 and D80 in KcsA, undergoing interactions with each other and SF residues mediated by water molecules. This network, in addition to specific T118 interactions, typically serves to maintain conductive

and nonconductive states with the SF, and it also stabilizes partially conductive conformations. These open states epitomize the flipped E71A mutant, with T118 and H122 mimicking the flexible nature of V76 and D80, respectively. In P2, the W67-D80 interaction is conserved in Y217 and D230 in the conductive conformation, but is primarily detached in the remaining conformations, with swelled S0 and S1 sites. This is consistent with the position of L228, where KcsA contains Y78, suggesting that the presence of this residue may destabilize this region.

To assess whether the dynamics we observed in the SF are unique to TWIK-1, we aligned multiple sequences constituting the SF and the surrounding environment (Fig. 6), and compared interactions that are known to stabilize the SF. In Kv1.2, which is representative of the voltage-gated K^+ channel family, residues D379 and W366 are integral and display exact conservation with D80 and W67 in KcsA, respectively. In Kir2.2, which is representative of the inward-rectifying K^+ channel family, a salt-bridge between E139 (conserved with E71) and R149 is responsible for maintenance of the conductive SF structure. This suggests that TWIK-1 cannot be likened to such channel families. With regard to other K2P channels, TRAAK and TREK-2 demonstrate highly conserved sequences in the pore region but diverge from TWIK-1 with regard to the T118, H122, and L228 positions, which contain I, N, and F residues, respectively. This is consistent with our proposal that the variability of the SF structure originates from these residues, and supports experimental evidence indicating that TWIK-1 is also atypical within the K2P family with regard to its conduction properties.

CONCLUSIONS

Throughout our computational study, we gained insights into the highly dynamic behavior of the TWIK-1 SF. Multiple K^+ ions can occupy alternate sites in the SF, bound in a typical cage-like manner to surrounding carbonyl atoms; however, we find the behavior of SF is disparate from that previously recorded.

**TWIK-1 (P1)**	FFASTVLSTTGYGHT
**TWIK-1 (P2)**	YFCFISLSTIGLGDY
**TRAAK (P1)**	FFSGTIITTIGYGNV
**TRAAK (P2)**	YFVIVTLTTVGFGDY
**TREK-2 (P1)**	FFAGTVITTIGYGNI
**TREK-2 (P2)**	YFVVVTLTTVGFGDF
**KcsA**	WWSVETATTVGYGDY
**Kv1.2**	WWAVVSMTTVGYGDM
**Kir2.2**	LFSIETQTTIGYGLR

FIGURE 6 Sequence alignment of the SF region with multiple K^+ channels. P1 and P2 denote the nonidentical domains observed in K2P channels. To see this figure in color, go online.

The divergence of the SF in comparison with other K^+ channels is exemplified at the S2 site, with diverse behaviors from both nonconserved P domains in the SF converging here. We identified multiple conformations in P1 that exhibited lateral expansion of the S2 and S3 sites, primarily depending on the conformational state of T118. Introduction of this residue into other K2P channels induced the dynamic-selectivity phenomenon observed in subphysiological conditions in native TWIK-1 channels; therefore, it is possible that such conformations may have varied selectivity properties. The dependence on the surrounding H-bond network, in particular the behavior of H122, raises the possibility that external stimuli, such as K^+ concentration and pH, can be translated to structural changes in the SF. In addition, we observed a widening at the extracellular mouth of the P2 domain, removing the upper sites in the SF. The protonation state of the residues of the selectivity was extensively tested. With the exception of a single simulation (with H122 protonated in the δ position and the unprotonated state of D230), ions were always observed to leave the binding site S2. Upon exclusion of ions from S2, we observed additional conformational states that physically blocked the SF, similar to what was observed in KcsA in low K^+ concentrations. Finally, in a simulation in which both H122 and D230 were in protonated forms and both ions were lost from the filter, the SF remained in an open conformation and all sites were filled with water molecules. TWIK-1 is the first channel, to our knowledge, to display such noninactivating characteristics in a computational simulation.

Overall, these results shed light on the conduction properties of TWIK-1, which exhibits low levels of activity in physiological conditions, yet a robust inward Na^+ current in subphysiological K^+ concentrations and upon acidification. The connection between such properties and the paradoxical depolarization of cardiomyocytes has potential implications for the development of channel-based therapies for associated cardiac disorders.

SUPPORTING MATERIAL

Three figures are available at http://www.biophysj.org/biophysj/supplemental/S0006-3495(16)30540-9.

AUTHOR CONTRIBUTIONS

V.O. performed the modeling and analyzed the data. V.O., S.F., and C.D. interpreted the data and wrote the manuscript. D.P. critically revised the manuscript.

ACKNOWLEDGMENTS

We thank ARCHER, the UK National Supercomputing Service (http://www.archer.ac.uk); the Hartree Centre; and the National Service for Computational Chemistry Software for providing computational resources.

This work was supported by the Biotechnology and Biological Sciences Research Council and Pfizer Neusentis.

REFERENCES

1. Enyedi, P., and G. Czirják. 2010. Molecular background of leak K+ currents: two-pore domain potassium channels. *Physiol. Rev.* 90: 559–605.
2. Sandoz, G., D. Douguet, …, F. Lesage. 2009. Extracellular acidification exerts opposite actions on TREK1 and TREK2 potassium channels via a single conserved histidine residue. *Proc. Natl. Acad. Sci. USA.* 106:14628–14633.
3. Kang, D., C. Choe, and D. Kim. 2005. Thermosensitivity of the two-pore domain K^+ channels TREK-2 and TRAAK. *J. Physiol.* 564:103–116.
4. Maingret, F., M. Fosset, …, E. Honoré. 1999. TRAAK is a mammalian neuronal mechano-gated K^+ channel. *J. Biol. Chem.* 274:1381–1387.
5. Lesage, F., F. Maingret, and M. Lazdunski. 2000. Cloning and expression of human TRAAK, a polyunsaturated fatty acids-activated and mechano-sensitive K^+ channel. *FEBS Lett.* 471:137–140.
6. Patel, A. J., E. Honoré, …, M. Lazdunski. 1999. Inhalational anesthetics activate two-pore-domain background K^+ channels. *Nat. Neurosci.* 2:422–426.
7. Es-Salah-Lamoureux, Z., D. F. Steele, and D. Fedida. 2010. Research into the therapeutic roles of two-pore-domain potassium channels. *Trends Pharmacol. Sci.* 31:587–595.
8. Miller, A. N., and S. B. Long. 2012. Crystal structure of the human two-pore domain potassium channel K2P1. *Science.* 335:432–436.
9. Brohawn, S. G., J. del Mármol, and R. MacKinnon. 2012. Crystal structure of the human K2P TRAAK, a lipid- and mechano-sensitive K^+ ion channel. *Science.* 335:436–441.
10. Brohawn, S. G., E. B. Campbell, and R. MacKinnon. 2014. Physical mechanism for gating and mechanosensitivity of the human TRAAK K^+ channel. *Nature.* 516:126–130.
11. Dong, Y. Y., A. C. W. Pike, …, E. P. Carpenter. 2015. K2P channel gating mechanisms revealed by structures of TREK-2 and a complex with Prozac. *Science.* 347:1256–1259.
12. Lesage, F., E. Guillemare, …, J. Barhanin. 1996. TWIK-1, a ubiquitous human weakly inward rectifying K^+ channel with a novel structure. *EMBO J.* 15:1004–1011.
13. Rajan, S., L. D. Plant, …, S. A. Goldstein. 2005. Sumoylation silences the plasma membrane leak K^+ channel K2P1. *Cell.* 121:37–47.
14. Feliciangeli, S., M. P. Tardy, …, F. Lesage. 2010. Potassium channel silencing by constitutive endocytosis and intracellular sequestration. *J. Biol. Chem.* 285:4798–4805.
15. Goldstein, S. A., L. A. Price, …, M. H. Pausch. 1996. ORK1, a potassium-selective leak channel with two pore domains cloned from Drosophila melanogaster by expression in Saccharomyces cerevisiae. *Proc. Natl. Acad. Sci. USA.* 93:13256–13261.
16. Ben-Abu, Y., Y. Zhou, …, O. Yifrach. 2009. Inverse coupling in leak and voltage-activated K^+ channel gates underlies distinct roles in electrical signaling. *Nat. Struct. Mol. Biol.* 16:71–79.
17. Chatelain, F. C., D. Bichet, …, F. Lesage. 2012. TWIK1, a unique background channel with variable ion selectivity. *Proc. Natl. Acad. Sci. USA.* 109:5499–5504.
18. Ma, L., X. Zhang, and H. Chen. 2011. TWIK-1 two-pore domain potassium channels change ion selectivity and conduct inward leak sodium currents in hypokalemia. *Sci. Signal.* 4:ra37.
19. Ma, L., Y. P. Xie, …, H. Chen. 2012. Silent TWIK-1 potassium channels conduct monovalent cation currents. *Biophys. J.* 102: L34–L36.
20. Ma, L., X. Zhang, …, H. Chen. 2012. Acid-sensitive TWIK and TASK two-pore domain potassium channels change ion selectivity and become permeable to sodium in extracellular acidification. *J. Biol. Chem.* 287:37145–37153.
21. Paice, B. J., K. R. Paterson, …, D. H. Lawson. 1986. Record linkage study of hypokalaemia in hospitalized patients. *Postgrad. Med. J.* 62:187–191.
22. Schaefer, T. J., and R. W. Wolford. 2005. Disorders of potassium. *Emerg. Med. Clin. North Am.* 23:723–747., viii–ix., (viii–ix.).
23. Schmidt, C., F. Wiedmann, …, D. Thomas. 2013. Class I antiarrhythmic drugs inhibit human cardiac two-pore-domain K^+ (K2 $_2$p) channels. *Eur. J. Pharmacol.* 721:237–248.
24. Aryal, P., F. Abd-Wahab, …, S. J. Tucker. 2014. A hydrophobic barrier deep within the inner pore of the TWIK-1 K2P potassium channel. *Nat. Commun.* 5:4377.
25. Aryal, P., F. Abd-Wahab, …, S. J. Tucker. 2015. Influence of lipids on the hydrophobic barrier within the pore of the TWIK-1 K2P channel. *Channels (Austin).* 9:44–49.
26. Aqvist, J., and V. Luzhkov. 2000. Ion permeation mechanism of the potassium channel. *Nature.* 404:881–884.
27. Fiser, A., and A. Sali. 2003. ModLoop: automated modeling of loops in protein structures. *Bioinformatics.* 19:2500–2501.
28. Li, H., A. D. Robertson, and J. H. Jensen. 2005. Very fast empirical prediction and rationalization of protein pKa values. *Proteins.* 61: 704–721.
29. Humphrey, W., A. Dalke, and K. Schulten. 1996. VMD: visual molecular dynamics. *J. Mol. Graph.* 14:33–38., 27–28.
30. Phillips, J. C., R. Braun, …, K. Schulten. 2005. Scalable molecular dynamics with NAMD. *J. Comput. Chem.* 26:1781–1802.
31. Klauda, J. B., R. M. Venable, …, R. W. Pastor. 2010. Update of the CHARMM all-atom additive force field for lipids: validation on six lipid types. *J. Phys. Chem. B.* 114:7830–7843.
32. Jorgensen, W. L., J. Chandrasekhar, …, M. L. Klein. 1983. Comparison of simple potential functions for simulating liquid water. *J. Chem. Phys.* 79:926–935.
33. Beglov, D., and B. Roux. 1994. Finite representation of an infinite bulk system: solvent boundary potential for computer simulations. *J. Chem. Phys.* 100:9050–9063.
34. Luo, Y., and B. Roux. 2010. Simulation of osmotic pressure in concentrated aqueous salt solutions. *J. Phys. Chem. Lett.* 1:183–189.
35. Darden, T., D. York, and L. Pedersen. 1993. Particle mesh Ewald: an N•log(N) method for Ewald sums in large systems. *J. Chem. Phys.* 98:10089–10092.
36. Verlet, L. 1967. Computer "experiments" on classical fluids. I. Thermodynamical properties of Lennard-Jones molecules. *Phys. Rev.* 159:98.
37. Miyamoto, S., and P. A. Kollman. 1992. SETTLE: an analytical version of the SHAKE and RATTLE algorithm for rigid water models. *J. Comput. Chem.* 13:952–962.
38. Feller, S. E., Y. Zhang, …, B. R. Brooks. 1995. Constant pressure molecular dynamics simulation: the Langevin piston method. *J. Chem. Phys.* 103:4613–4621.
39. Martyna, G. J., D. J. Tobias, and M. L. Klein. 1994. Constant pressure molecular dynamics algorithms. *J. Chem. Phys.* 101:4177–4189.
40. Hoshi, T., and C. M. Armstrong. 2013. C-type inactivation of voltage-gated K^+ channels: pore constriction or dilation? *J. Gen. Physiol.* 141:151–160.
41. Kiss, L., J. LoTurco, and S. J. Korn. 1999. Contribution of the selectivity filter to inactivation in potassium channels. *Biophys. J.* 76:253–263.
42. Korn, S. J., and S. R. Ikeda. 1995. Permeation selectivity by competition in a delayed rectifier potassium channel. *Science.* 269:410–412.
43. Wang, Z., X. Zhang, and D. Fedida. 2000. Regulation of transient Na+ conductance by intra- and extracellular K+ in the human delayed rectifier K^+ channel Kv1.5. *J. Physiol.* 523:575–591.
44. Starkus, J. G., L. Kuschel, …, S. H. Heinemann. 1997. Ion conduction through C-type inactivated Shaker channels. *J. Gen. Physiol.* 110:539–550.

45. Starkus, J. G., L. Kuschel, …, S. H. Heinemann. 1998. Macroscopic Na+ currents in the “Nonconducting” Shaker potassium channel mutant W434F. *J. Gen. Physiol.* 112:85–93.

46. Gang, H., and S. Zhang. 2006. Na^+ permeation and block of hERG potassium channels. *J. Gen. Physiol.* 128:55–71.

47. Doyle, D. A., J. Morais Cabral, …, R. MacKinnon. 1998. The structure of the potassium channel: molecular basis of K^+ conduction and selectivity. *Science.* 280:69–77.

48. Cordero-Morales, J. F., L. G. Cuello, …, E. Perozo. 2006. Molecular determinants of gating at the potassium-channel selectivity filter. *Nat. Struct. Mol. Biol.* 13:311–318.

49. Zhou, Y., J. H. Morais-Cabral, …, R. MacKinnon. 2001. Chemistry of ion coordination and hydration revealed by a K^+ channel-Fab complex at 2.0 A resolution. *Nature.* 414:43–48.

50. Domene, C., and S. Furini. 2009. Dynamics, energetics, and selectivity of the low-K^+ KcsA channel structure. *J. Mol. Biol.* 389:637–645.

51. Domene, C., M. L. Klein, …, M. Parrinello. 2008. Conformational changes and gating at the selectivity filter of potassium channels. *J. Am. Chem. Soc.* 130:9474–9480.

52. Cuello, L. G., V. Jogini, …, E. Perozo. 2010. Structural basis for the coupling between activation and inactivation gates in K^+ channels. *Nature.* 466:272–275.

53. Cuello, L. G., V. Jogini, …, E. Perozo. 2010. Structural mechanism of C-type inactivation in K^+ channels. *Nature.* 466:203–208.

54. Furini, S., O. Beckstein, and C. Domene. 2009. Permeation of water through the KcsA K^+ channel. *Proteins.* 74:437–448.

55. Hoomann, T., N. Jahnke, …, P. Pohl. 2013. Filter gate closure inhibits ion but not water transport through potassium channels. *Proc. Natl. Acad. Sci. USA.* 110:10842–10847.

56. Raghuraman, H., S. M. Islam, …, E. Perozo. 2014. Dynamics transitions at the outer vestibule of the KcsA potassium channel during gating. *Proc. Natl. Acad. Sci. USA.* 111:1831–1836.

57. van der Kamp, M. W., R. D. Schaeffer, …, V. Daggett. 2010. Dynameomics: a comprehensive database of protein dynamics. *Structure.* 18:423–435.

58. Ostmeyer, J., S. Chakrapani, …, B. Roux. 2013. Recovery from slow inactivation in K^+ channels is controlled by water molecules. *Nature.* 501:121–124.

59. Cordero-Morales, J. F., V. Jogini, …, E. Perozo. 2007. Molecular driving forces determining potassium channel slow inactivation. *Nat. Struct. Mol. Biol.* 14:1062–1069.

60. Cordero-Morales, J. F., V. Jogini, …, E. Perozo. 2011. A multipoint hydrogen-bond network underlying KcsA C-type inactivation. *Biophys. J.* 100:2387–2393.

Biophysical Journal, Volume *111*

Supplemental Information

Exploring the Dynamics of the TWIK-1 Channel

Victoria Oakes, Simone Furini, David Pryde, and Carmen Domene

SUPPLEMENTARY INFORMATION

Exploring the dynamics of the TWIK-1 channel

Victoria Oakes,[1] Simone Furini,[2] David Pryde,[3] Carmen Domene[1,4]

[1]Department of Chemistry, King's College London, Britannia House, 7 Trinity Street, London, SE1 1DB, UK.
[2]Department of Medical Biotechnologies, University of Siena, viale Mario Bracci 16, I-53100, Siena, Italy
[3]Worldwide Medicinal Chemistry, Pfizer Pfizer Neuroscience and Pain Research Unit, Portway Building, Granta Park, Great Abington, Cambridge CB21 6GS, UK
[4]Chemistry Research Laboratory, Mansfield Road, University of Oxford, Oxford, OX1 3TA, UK.

[1]Corresponding author: carmen.domene@kcl.ac.uk

Tel: +44 - (0) 2078483868

Figure S1. Root mean square deviation (RMSD) of the backbone of the transmembrane helices of TWIK-1 in simulations: (A) HSE, (B) HSEP, (C) HSD, (D) HSDP, (E) HSP and (F) HSPP.

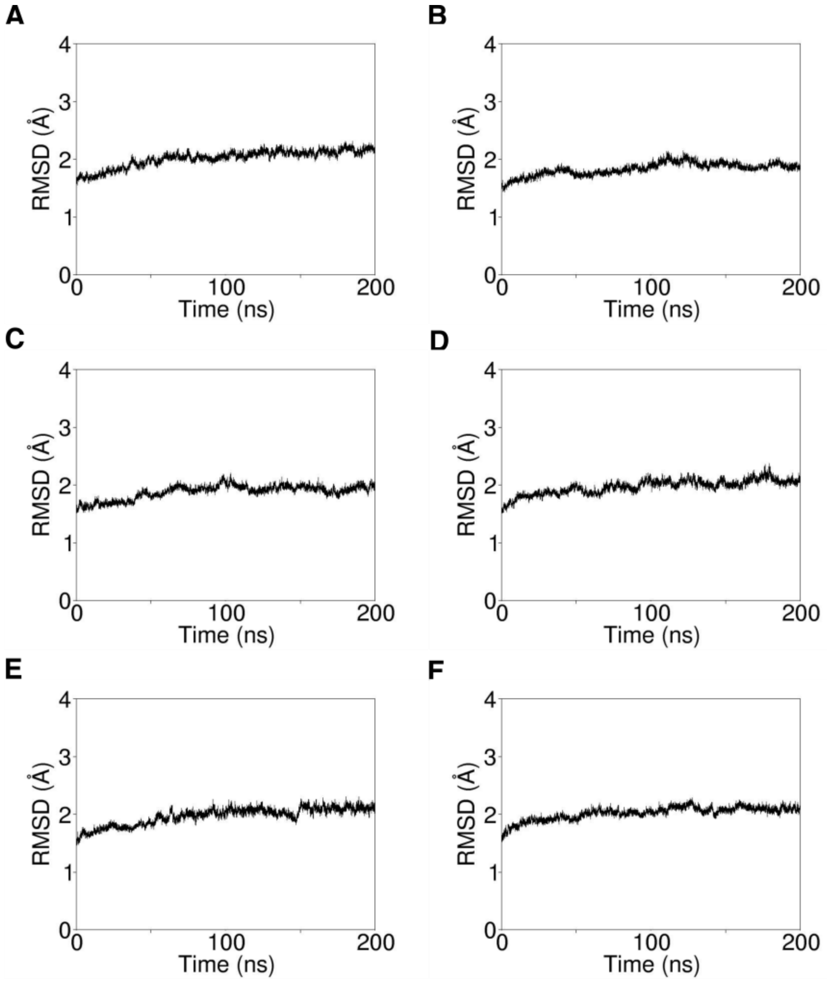

Figure S2. Root mean square deviation (RMSD) of the extracellular cap corresponding to residues S80 to D103 in simulations: (A) HSE, (B) HSEP, (C) HSD, (D) HSDP, (E) HSP and (F) HSPP.

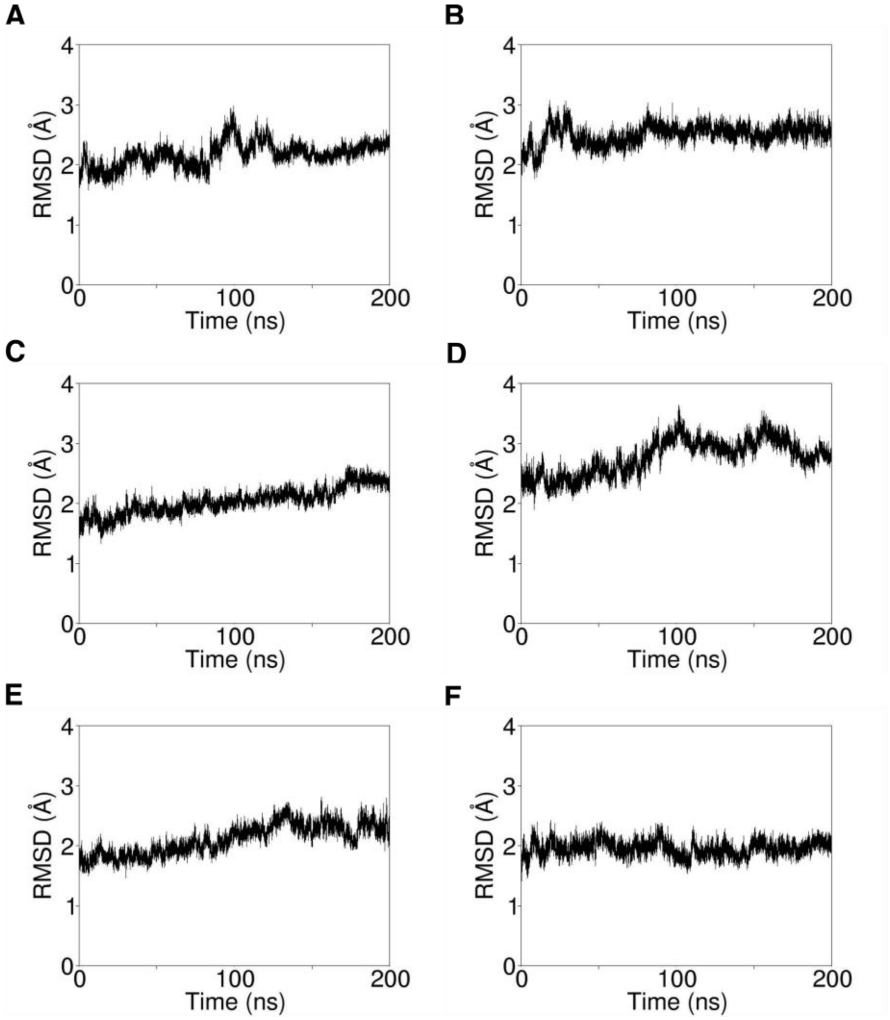

Figure S3. Representative snapshots of each ion configuration throughout simulations (A) HSE, (B) HSEP, (C) HSD, (D) HSDP, (E) HSP and (F) HSPP. The selectivity filter residues T117 to G121 and T225 to G229 are displayed in licorice representation, with Van der Waals spheres representing ions and water molecules, with oxygen, nitrogen, carbon, sodium and potassium atoms shown in red, blue, cyan, yellow and orange respectively.

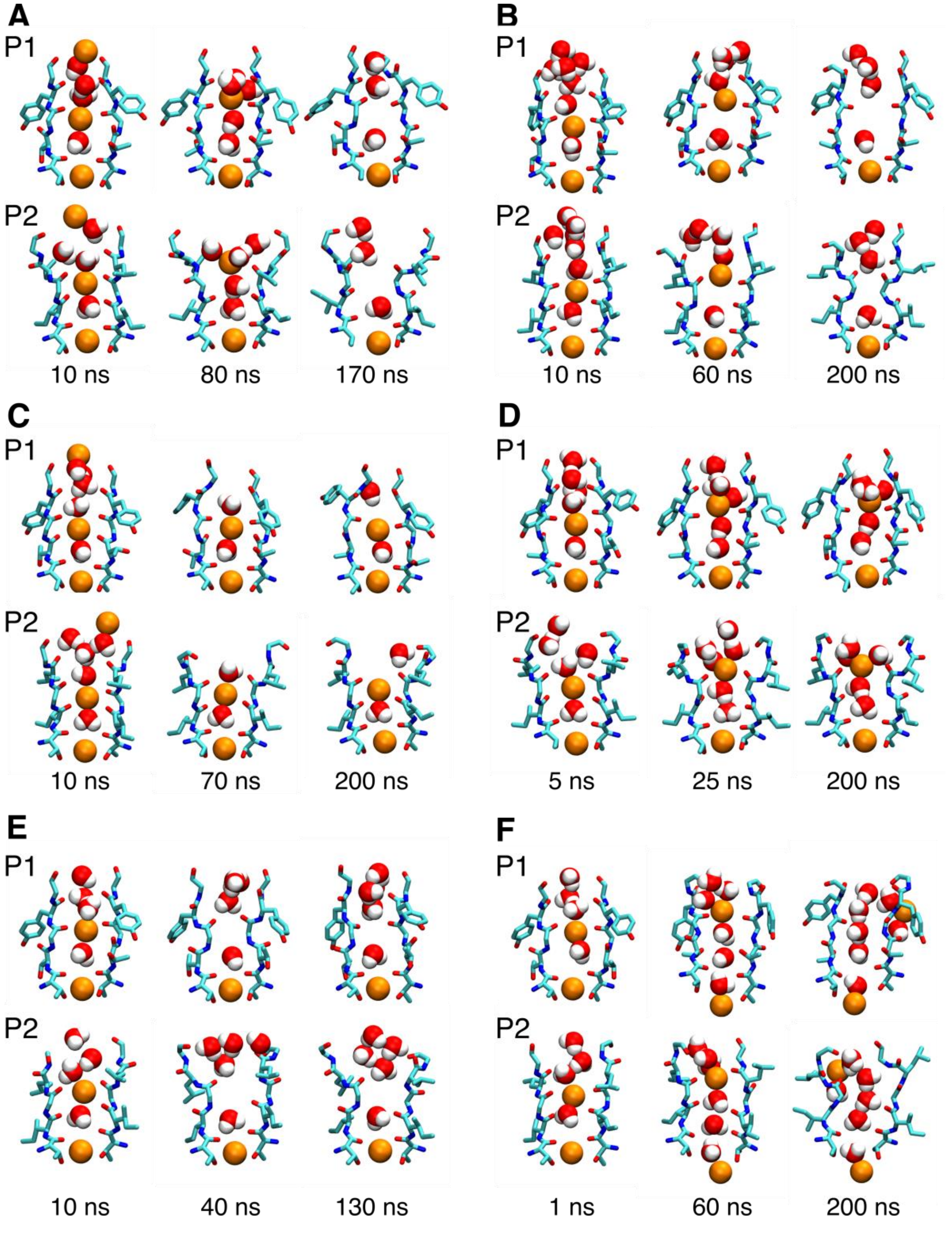

Supplement: Document S2. Article plus Supporting Material [file mmc2.pdf]
